# An Unusual Case of Dilated Cardiomyopathy in Wegner’s Granulomatosis

**DOI:** 10.7759/cureus.25975

**Published:** 2022-06-15

**Authors:** Ram Chandra Khatri Chhetri, Shrey Gole, Arvin Junn P Mallari, Aman Dutta, Farah Zahra

**Affiliations:** 1 Internal Medicine, Chicago Medical School Internal Medicine Residency Program at Northwestern McHenry Hospital, McHenry, USA

**Keywords:** dilated cardiomyopathy (dcm), anca-associated vasculitis, heart failure, granulomatosis with polyangiitis (gpa), wegner’s granulomatosis

## Abstract

A 33-year-old male presented to the emergency with cough, hemoptysis, and shortness of breath. He was on steroids for suspected Still’s disease due to arthralgias and fever prior to presentation to the emergency. He developed sudden hypoxic respiratory failure and required mechanical ventilation. The initial imaging studies of the chest including computed tomography (CT) of the chest showed marked diffuse central and basilar predominant opacities with associated smooth septal thickening. Furthermore, the patient’s creatinine, troponin, B-type natriuretic peptide (BNP), rheumatoid factor, and D-dimer were elevated. Vasculitis workup, bronchoscopy, and echocardiogram were performed. The echocardiogram revealed severely decreased left ventricular systolic function with an ejection fraction of 24% with dilated left ventricle. The electrocardiogram did not show any findings of acute ischemia. He was started on pulse dose steroid and dobutamine drip along with intermittent diuresis. The patient was successfully extubated after two days of mechanical ventilation. He was started on cyclophosphamide in the hospital. Dobutamine was discontinued. He was moved to the general medical floor as his oxygenation improved, but later at night, he developed respiratory failure and required a bumetanide drip. The cytoplasmic antineutrophil cytoplasmic antibodies (C-ANCA) (anti-PR-3 antibody) came back positive with titer >1:40, so Wegner’s granulomatosis was diagnosed. He received three sessions of plasmapheresis. The patient’s kidney function improved significantly, and the bumetanide drip was transitioned to intravenous pushes. His oxygenation improved significantly with saturations of 92% on room air. The patient was discharged on steroid, Bactrim, and systolic heart failure medications to follow up with rheumatology, nephrology, pulmonology, and cardiology in the office. Due to insurance issues, his outpatient care was delayed significantly. The patient followed up with rheumatology after two months and has been planned for rituximab induction and to continue steroid along with Bactrim. This case is worth reporting because it describes dilated cardiomyopathy (DCM) as a cardiac manifestation of Wegner’s granulomatosis. Early cardiac evaluation should be incorporated into the management of the patient suspected of Wegner’s granulomatosis.

## Introduction

Granulomatosis with polyangiitis (GPA), also known as Wegner’s granulomatosis, is a rare form of antineutrophil cytoplasmic antibody (ANCA)-associated vasculitis of the small- and medium-sized blood vessels affecting mainly the upper and lower respiratory tracts as well as the kidneys. The prevalence of GPA is 30.3 cases per million persons [1]. Pulmonary manifestations include cavitary lesions, pulmonary hemorrhage, and fibrosis in chronic cases. Cardiac involvement in Wegner’s granulomatosis occurs in 6%-44% of cases [2-4]. Pericarditis and coronary arteritis are the most common cardiac manifestations, but left ventricular global systolic dysfunction, conduction abnormalities, and pericardial effusion have also been described [4,5]. Only four case reports of dilated cardiomyopathy (DCM) associated with GPA have been described [6-8]. The patients described in these reports had left ventricular systolic dysfunction with reduced ejection fraction.

## Case presentation

A 33-year-old male with a past medical history of rheumatic fever as a child presented to the emergency department (ED) with two days of cough, hemoptysis, and shortness of breath associated with subjective fever and chills. He denied orthopnea, paroxysmal nocturnal dyspnea, lower extremity edema, chest pain, or palpitations. His symptoms had begun four months prior with fatigue, generalized myalgias, arthralgias, and bilateral distal lower extremity weakness causing difficulty ambulating. He also reported a 100 lb weight loss in the previous four months. However, he denied proximal muscle weakness, rashes, changes in skin texture, dysphagia, morning stiffness, or dysuria. He had been started on prednisone 60 mg daily by his primary care physician three months prior to presentation, which was being tapered down, with him being on 20 mg daily at the time of presentation. His family history was significant for rheumatoid arthritis and fibromyalgia in his sister.

**Figure 1:**
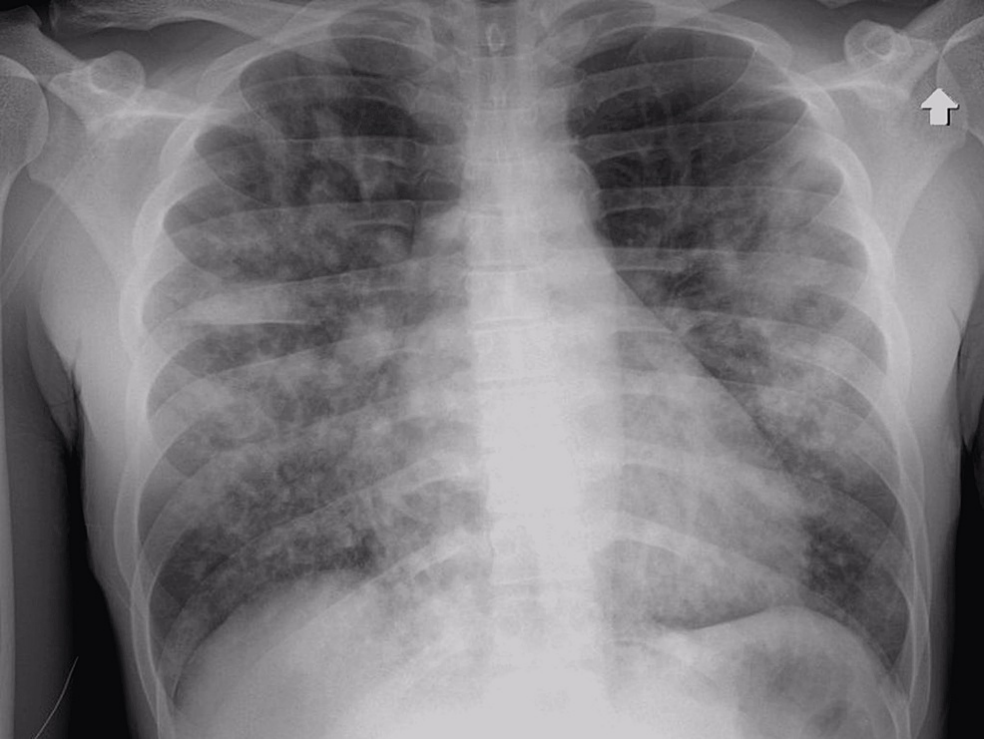
Chest radiography showing diffuse multifocal nodular opacities

**Figure 2:**
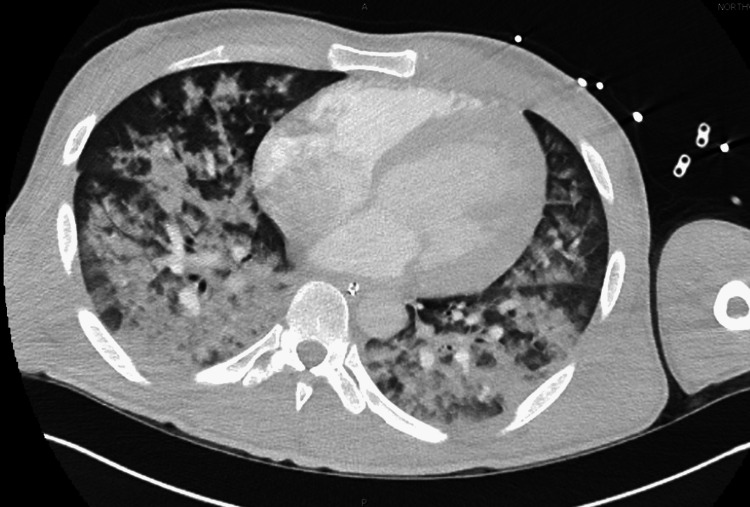
Computed tomography with angiography of the chest showing diffuse multifocal opacities

In the ED, his oxygen saturation was low at 65% on high-flow oxygen, blood pressure was 110/60 mmHg, and heart rate was 112/minute. The patient was coughing up bright red blood. The patient was intubated and transferred to the ICU. On physical examination, he had inspiratory and expiratory crackles. Laboratory evaluation revealed increased leukocyte count of 17.5, elevated troponin of 0.41 ng/mL (reference: <0.10 ng/mL), elevated B-type natriuretic peptide (BNP) of 3,080 pg/mL (reference: <101 pg/mL), elevated D-dimer of >5,000 ng/mL D-DU (reference: 0-230 ng/mL D-DU), and elevated lactic acid of 2.4 mMol/L (reference: 0.7-1.9 mMol/L). His COVID-19 PCR had been negative on multiple occasions. Creatinine was elevated at 1.83 mg/dL (reference: 0.60-1.30 mg/dL). INR was 1.3. Imaging studies with chest radiography (Figure 1) and computed tomography (CT) angiography of the chest for pulmonary embolism (Figure 2) showed marked diffuse central and basilar predominant opacities with associated small septal thickening.

**Figure 3:**
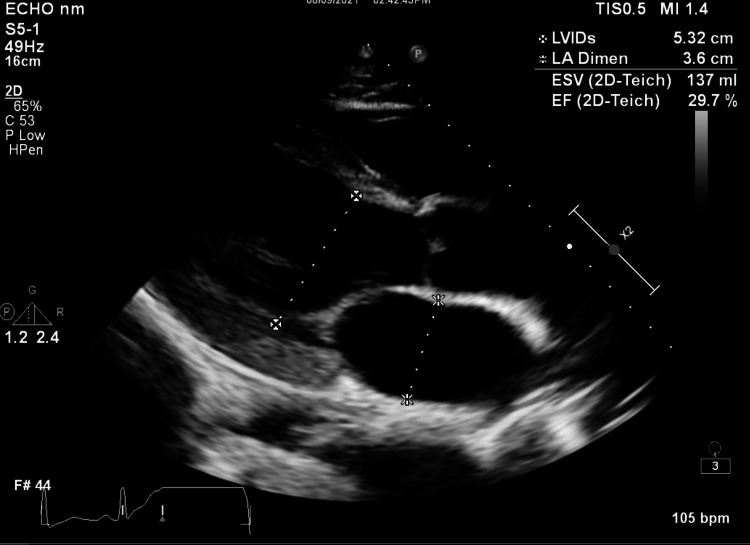
Transthoracic echocardiogram showing elevated systolic left ventricle internal dimension with left ventricular dilation

In the ICU, tests including ANCA, anti-SSA/SSB, anti-dsDNA, anti-Smith antibody, anti-glomerular basement antibody (anti-GBM), C3, and C4 were sent. Bronchoscopy was done, and the bronchoalveolar lavage studies revealed predominantly benign bronchial epithelial cells, macrophages, and degenerative cells with insignificant lymphocytes of 12% and neutrophils of 75%. Furthermore, the thyroid function test was normal. The patient was started on a pulse dose of methylprednisolone 1 g/day for three days. Transthoracic echocardiogram showed left ventricular dilation (Figure 3) without left ventricular hypertrophy, severely decreased left ventricular systolic function with an ejection fraction of 24%, and normal right ventricular systolic function. Creatinine was slowly rising despite adequate support and resuscitation. The patient was started on a dobutamine drip. His anti-GBM was <1. Rheumatoid factor titers were very high at 1:320. ESR was 86 mm/hour, and CRP was 9.7 mg/L (reference: <0.3 mg/L).

The patient’s respiratory status improved, and he was extubated on day 3 of admission. He was started on cyclophosphamide 1.5 g monthly with a plan to switch to azathioprine as maintenance. Dobutamine drip was discontinued the next day, and steroid therapy was switched to prednisone 1 mg/kg daily. However, his respiratory status again deteriorated, and he was started on a bumetanide drip. The vasculitis workup revealed elevated C-ANCA titers (>1:40) and anti-proteinase-3 antibody positive with 3.7, suggesting GPA diagnosis. As his respiratory status was worsening, he underwent three sessions of plasmapheresis, with significant improvement. The patient was discharged, and outpatient C-ANCA titers have been negative. He is currently doing well on rituximab. The patient has not followed up with cardiology for cardiac magnetic resonance imaging (MRI) and cardiac catheterization.

## Discussion

GPA can involve multiple systems and present with a variety of symptoms. The prevalence of cardiac involvement is 6%-44% [2-4]. Common cardiac manifestations are pericarditis, myocarditis, and conduction abnormalities [5,9]. DCM associated with GPA is very rare and has been reported in a few case reports since GPA was described in 1936 by Wegner [4,7,8,10]. The exact mechanism of cardiac failure is unknown.

When paired with tests (C-ANCA, ESR, and CRP), imaging studies such as cardiac MRI have revolutionized the way GPA complications can be determined. However, tissue diagnosis remains the gold standard. In this case, GPA was associated with acute systolic dysfunction, pulmonary hemorrhage, positive C-ANCA (anti-PR-3 antibody), and acute kidney injury (AKI). The temporal relation of acute-onset systolic dysfunction and kidney injury in a patient without a history of cardiac and kidney disease history proves that the acute clinical manifestations are related to GPA. To our knowledge, this is the fifth case of acute DCM associated with GPA. Clinicians should be aware that GPA can present with acute DCM, and cardiac evaluation should be prompted early in the management of patients with GPA.

## Conclusions

We would like to inform the clinicians from this case report about the unusual presentation of pulmonary hemorrhage in a young patient that led to the diagnosis of GPA. Furthermore, this was complicated by systolic cardiac dysfunction and AKI, which raised the suspicion of a multiorgan vasculitic process. Hence, cardiac evaluation should be prompted early in the management of patients with GPA. Cardiac MRI paired with other diagnostic serologic tests for GPA can assist in prompt diagnosis and manage the complications from systolic dysfunction.
